# The development and characterisation of a bacterial artificial chromosome library for *Fragaria vesca*

**DOI:** 10.1186/1756-0500-2-188

**Published:** 2009-09-23

**Authors:** Julio Bonet, Elena Lopez Girona, Daniel J Sargent, Monica C Muñoz-Torres, Amparo Monfort, Albert G Abbott, Pere Arús, David W Simpson, Jahn Davik

**Affiliations:** 1 IRTA. Centre de Recerca en Agrigenòmica CSIC-IRTA-UAB, 38348 Cabrils, Spain; 2 East Malling Research (EMR), New Road, East Malling, Kent ME19 6BJ, UK; 3 Clemson University, Department of Genetics and Biochemistry, Clemson, SC 29634 USA; 4 Bioforsk Midt-Norge, Kvithamar, N-7500 Stjordal, Norway; 5 Current address : Georgetown University, Department of Biology, Washington, DC 20057 USA

## Abstract

**Background:**

The cultivated strawberry *Fragaria ×ananassa *is one of the most economically-important soft-fruit species. Few structural genomic resources have been reported for *Fragaria *and there exists an urgent need for the development of physical mapping resources for the genus. The first stage in the development of a physical map for *Fragaria *is the construction and characterisation of a high molecular weight bacterial artificial chromosome (BAC) library.

**Methods:**

A BAC library, consisting of 18,432 clones was constructed from *Fragaria vesca *f. *semperflorens *accession 'Ali Baba'. BAC DNA from individual library clones was pooled to create a PCR-based screening assay for the library, whereby individual clones could be identified with just 34 PCR reactions. These pools were used to screen the BAC library and anchor individual clones to the diploid *Fragaria *reference map (FV×FN).

**Findings:**

Clones from the BAC library developed contained an average insert size of 85 kb, representing over seven genome equivalents. The pools and superpools developed were used to identify a set of BAC clones containing 70 molecular markers previously mapped to the diploid *Fragaria *FV×FN reference map. The number of positive colonies identified for each marker suggests the library represents between 4× and 10× coverage of the diploid *Fragaria *genome, which is in accordance with the estimate of library coverage based on average insert size.

**Conclusion:**

This BAC library will be used for the construction of a physical map for *F. vesca *and the superpools will permit physical anchoring of molecular markers using PCR.

## Research Hypothesis

The strawberry species *Fragaria ×ananassa *is a member of the Rosaceae, and is one of the most economically-important soft-fruit species. However, it is an allo-octoploid species (2*n *= 8*x *= 56), which grants it genetically complex status. *Fragaria vesca *is a diploid (2*n *= 2*x *= 14) relative of the cultivated strawberry, that has been demonstrated to be the closest extant diploid to the ancestor of the cultivated octoploid strawberry [1,2]. *F. vesca *has a genome size of approximately 206 Mb/C [3], a short reproductive cycle and facile vegetative and seed propagation, as well as amenability to genetic transformation. These features make it an ideal model organism for forward and reverse genetics studies in *Fragaria*.

There currently exists a well characterised, saturated genetic linkage map for diploid *Fragaria *developed from an interspecific cross between *F. vesca *and another closely-related diploid species, *F. bucharica *(FV×FB). Comparisons between this map and maps of the cultivated strawberry have shown that the diploid genome of *F. vesca *is essentially completely collinear with the four genomes that make up the allooctoploid genome of the cultivated strawberry [4]. Despite a growing body of molecular genetics resources, to date, very few structural genomic resources have been reported for the genus *Fragaria *[5]. High molecular weight DNA libraries are an essential resource for genomic investigations, including positional cloning of genes of economic importance, such as the *fw*2.2 QTL in tomato [6]; for the determination of gene structure and function [7]; for region-targeted marker development [8]; comparative genome analysis [9]; genome sequencing [10]; and the construction of physical maps [11]. Due to their stability, ability to maintain relatively high molecular weight genomic DNA inserts (over 150 kb), and their ease of handling and propagation, bacterial artificial chromosome (BAC) libraries have become the vector of choice for the development of DNA libraries in many plant species, including *Malus*, *Prunus *and *Rosa *in the Rosaceae [7,12,13].

In this paper, we report the development of a high molecular weight DNA BAC library from a diploid strawberry *F. vesca *f. *semperflorens *cv. 'Ali Baba' as a resource for the construction and characterisation of a physical map for diploid *Fragaria *and for positional cloning efforts. We have calculated that the library contains an average insert size of 85 kb and thus represents an estimated 7.6× coverage of the *Fragaria *genome. To allow rapid characterisation of this library, we developed a set of PCR pools and superpools from the library and identified 102 BAC clones containing 70 genetically mapped molecular markers (ten per linkage group), distributed throughout the seven diploid *Fragaria *linkage groups. These results demonstrate the efficiency of our PCR pools and superpools and provide a foundation for the development of physical mapping resources for the genus.

## Methods

### High molecular weight DNA preparation

A total of 20 g of young leaf tissue from the *Fragaria vesca *f. *semperflorens *cultivar 'Ali Baba' that had been given 72 h dark treatment at 16°C, was snap-frozen in liquid nitrogen and ground to a fine powder. The powdered tissue was then transferred to a 1 litre flask with 200 ml of nucleic isolation buffer ((NIB) 10 mM Tris, 10 mM EDTA, 100 mM KCl, 500 mM sucrose, 4 mM spermidine, 1 mM spermine, 2% w/v PVP MW 40000, 0.13% DIECA, 0.1% ascorbic acid, and 0.2% β-mercaptoethanol (BME)). The homogenate was shaken gently for 30 minutes at room temperature, before filtering through 2 layers of cheesecloth and 2 layers of Miracloth into a clean flask. A 1/20^th ^volume of lysis buffer (10% Triton-X 100 in NIB) was added and the flask was incubated on ice for 10 min, gently swirling every other minute.

Aliquots were transferred to 50 ml falcon tubes and the nuclei spun down at 1800 g for 15 min at 4°C. The supernatant was discarded and the nuclei resuspended in approximately 1 ml of NIB. Nuclei from two tubes were then combined, NIB was added to fill the tube and it was centrifuged at 1800 g for a further 15 min at 4°C. This procedure was repeated until all nuclei had been combined in a single tube. The supernatant was removed and 2 ml of NIB (without BME) was added along with an equal volume of 1.5% low-melting temperature agarose. The mix was then allowed to solidify in plug moulds at room temperature.

### Treatment of the plugs

After solidification, the plugs were treated twice with EPS buffer (0.5 M EDTA pH9.2, 1.0% sarcosyl, 0.1% BME, 1 mg/ml proteinase K) in 50 ml falcon tubes and incubated at 50°C for 24 h. Subsequently, the plugs were rinsed twice in sterile water with 0.1% BME before washing twice in 1 mM PMSF, 10 mM Tris, 10 mM EDTA at room temperature. The plugs were then rinsed twice with 0.1% BME followed by a double wash for 60 min each time with 10 mM Tris, 1 mM EDTA.

### Partial digestion and size selection of the high molecular weight DNA

Three units of *Hind*III was used to digest eight DNA plugs. Size selection of *Fragaria *DNA was performed with two consecutive rounds of pulse field gel electrophoresis (PFGE), conducted at 12°C, with 1 - 40 sec switch time, 6 V/cm, 120 deg angle for 18 hrs on a CHEF-DR-II system (Bio-Rad, Hercules, CA, USA). Fragments between 100 and 300 Kb in size were cut out and divided into three equal sections. A second size selection was performed on the three sections but the DNA was allowed to electrophorese into low melting point agarose with 3 - 5 sec switch time. Subsequently, fragments in the 100 to 300 Kb size range were excised from the gel and cut into cubes for electro elution using a Bio-Rad electro eluter (Bio-Rad, Hercules, CA, USA), following the manufacturer's protocol.

### Ligation and transformation

The eluted DNA was ligated into the pIndigoBAC536 vector [14] using a target DNA to vector ratio of 10:1 and transformed into ElectroMAX™ DH10B™ *Escherichia coli *cells (Invitrogen, Carlsbad, CA, USA) by electroporation following manufacturer's suggestions. Transformations were plated on Q-trays with LB-agar medium containing X-gal, IPTG and cholramphenicol. The Colonies were grown overnight and picked with a Genetix robot (Genetix, New Milton, UK) following the manufacturer's operating guidelines. A total of 18,432 colonies were picked and arrayed into forty-eight 384-well plates.

### Estimation of BAC clone insert sizes

Thirty-eight randomly-selected BAC clones were grown for 16 h at 37°C in 5 ml of LB containing 12.5 μg/mL chloramphenicol. BAC DNA was extracted from 38 clones following a standard alkaline lysis miniprep technique [15], digested with *Not*I and insert sizes were estimated against the λ size standard using PFGE in a CHEF system (Bio-Rad, Hercules, CA, ISA).

### Pool and superpool development

The 18,432 clone library, contained in fourty-eight 384-well plates, was condensed into fourty-eight 96-well plates (4,608 cultures), with each culture containing four adjacent clones from the original 384-well plates. BAC colony cultures were then pooled in three separate orientations from the 4,608 culture pools - 48 plate pools, 48 column pools and 32 row pools containing the entire library. The DNA was isolated from the pooled cultures following a standard alkaline lysis miniprep technique [15]. Subsequently, DNA from each of six plates (2,304 BAC clones each) was pooled to create eight superpools for prescreening of the library.

### Library screening and identification of BAC clones containing markers from the diploid *Fragaria *reference map

A set of 70 molecular markers (47 SSRs, one SNP, two EST-SSRs, nine ESTs, and 11 gene specific STS markers) evenly spaced throughout the seven linkage groups of the diploid *Fragaria *reference map (ten per linkage group) [16,17] were selected for PCR analysis of pooled BAC DNA (see Additional file 1). PCR reactions were performed using the DNA superpools as template, followed by a second round of PCR with the DNA plate, row and column pools corresponding to the positive DNA superpools. Reactions were performed following touch-down conditions from Sargent et al. [18] from 55-50°C and visualised following agarose gel electrophoresis. The four clones identified by each positive PCR were then grown overnight and used as template for PCR following the procedures described above, and single positive BAC clones were identified following electrophoresis and visualization over UV light.

## Results and discussion

### BAC clone insert sizes, chloroplast content and library genome coverage

The *Fragaria *BAC library created is composed of 18,432 BAC clones arrayed into forty-eight 384-well microplates. Of the 38 randomly-selected BAC clones analysed to assess the average insert size of the library, all but one (97%) contained inserts. The insert sizes of the clones ranged from 35 kb to 145 kb, with an average insert size of 85 kb, which is comparable with BAC libraries developed for other Rosaceous crops such as peach (70 kb; [8]), rose (102 kb; [12]) and apple (110 kb; [7]). The *F. vesca *genome size has been estimated to be in the region of 206 Mb [3], and thus, a total of 18,432 clones with an average insert size of 85 kb represents coverage of approximately 7.6× the *F. vesca *genome. The library has been denoted CUFvAB (Clemson University *F*. *vesca *'Ali Baba') for ease of reference, and each clone is identified by its plate number and 384-well cell reference i.e. CUFvAB04A01 refers to clone A01 on plate 4.

### PCR analysis of BAC library pools and superpools

All markers screened on the superpools of the *F. vesca *'Ali Baba' BAC library amplified a PCR product of the expected size in relation to amplicons generated from *F. vesca *genomic DNA, and when single positive colonies were identified, all contained the expected PCR marker, indicating no cross-contamination in the library. The results of the pool and superpool screens suggests the BAC library represents between 4× and 10× coverage of the diploid *Fragaria *genome, which is in accordance with the estimate of the library coverage based on average insert size. The BACs to which the markers are anchored are detailed in Additional file 1. In total, 102 BAC clones anchored to 70 genetically-mapped molecular markers (47 SSRs, one SNP, two EST-SSRs, nine ESTs, and 11 gene specific STS markers) that were evenly distributed (ten per linkage group) across the seven linkage groups of the diploid *Fragaria *reference map were identified and verified in this investigation.

## Conclusion

We have developed a BAC library consisting of 18,432 clones from DNA of the *F. vesca *f. *semperflorens *accession 'Ali Baba' with an average insert size of 85 Kb. At an effective 7.6× coverage of the *Fragaria *haploid genome, this library provides a good basis for the development of further genomics resources for *Fragaria*. This is the first high molecular weight genomic library reported for the genus and as such, is a valuable resource for genomics studies in *Fragaria*. Despite the initial time investment in the development of the library pools and superpools being relatively high, the availability of a PCR-based library screen means large numbers of markers, including SSRs, can now be rapidly associated with individual BAC clones in an efficient and cost-effective manner as has been demonstrated here. To construct physical maps, BAC libraries consisting of high molecular weight genomic DNA inserts are an essential prerequisite. The library presented here will enable the development of a physical map resource for *Fragaria *and the associated PCR pools and superpools will permit the rapid anchoring of genetic markers to a physical map framework. The BAC library will also prove to be a valuable tool for the continued development of linkage maps for the cultivated strawberry (*F. ×ananassa*). The identification of BAC clones anchored to regions of the diploid *Fragaria *reference map in which there is a current paucity of markers on the maps of the cultivated strawberry [4,19], will permit the targeted development of polymorphic markers in those regions, as has been previously successfully demonstrated for other species such as peach [8].

## Library availability

The library reported here and the associated PCR pools and superpools are available on a cost recovery basis from the communicating author.

## Competing interests

The authors declare that they have no competing interests.

## Authors' contributions

JB developed the pools and superpools, screened the library for genetic markers and drafted the manuscript; DJS developed the pools and superpools, screened the library for genetic markers and drafted the manuscript; MCMT developed the BAC library and critically reviewed the manuscript; AM developed the pools and superpools and critically reviewed the manuscript; AGA conceived the idea for a library, planned the experiments, managed the project at Clemson and critically reviewed the manuscript; PA planned the experiments, managed the project at Cabrils and drafted the manuscript; DWS managed the experiments at East Malling and critically reviewed the manuscript; JD developed the BAC library, screened the library for cpDNA contamination and drafted the manuscript. All authors read and approved the final manuscript.

## Supplementary Material

Additional file 1 ***Fragaria *molecular markers and associated BAC clones**. A list of the 70 genetic markers and the 102 BAC clones they identified through PCR screening is provided. The table also lists the marker type, position on the diploid *Fragaria *reference map FV×FN of Sargent et al. [20] and the primer sequences. Click here for file
