# Supplementary material for: The development and characterisation of a bacterial artificial chromosome library for Fragaria vesca
Source: BMC Res Notes. 2009 Sep 23;2:188. doi: 10.1186/1756-0500-2-188 (PMC2754993; doi:10.1186/1756-0500-2-188)
Supplement: Additional file 1 — Fragaria molecular markers and associated BAC clones. A list of the 70 genetic markers and the 102 BAC clones they identified through PCR screening is provided. The table also lists the marker type, position on the diploid Fragaria reference map FV×FN of Sargent et al. [20] and the primer sequences. [file 1756-0500-2-188-S1.DOC]

***Fragaria* molecular markers and associated BAC clones.**

The table lists the 70 genetic markers and the 102 BAC clones they identified through PCR screening. The table also lists the marker type, position on the diploid *Fragaria* reference map FV×FN of Sargent et al. [1] and the primer sequences. The final five alphanumeric characters of the BAC clone identifier refer to the plate number and row and column on each of the 48 plates in the library (i.e. CUFvAB31A04 refers to cell A04 on plate 31).

| Locus name | Marker type | Primer sequences 5’ – 3’ | FV×FN Linkage group | Map position | BAC clones | Ref† |
| --- | --- | --- | --- | --- | --- | --- |
| EMFn049 | SSR | F: GCTAGCGTTCAAATTAAGACCA | FG1 | 6.3 | CUFvAB19G11 | 1 |
|  |  | R: GGTATCGGATTGAGGAAATCAA |  |  |  |  |
| UDF002 | SSR | F: TATGGCCAGGATTGTTTGCT | FG1 | 15 | CUFvAB01I20 | 2 |
|  |  | R: TAGGAGGAGGCGTTGAAATG |  |  |  |  |
| EMFn136 | SSR | F: TTTCTCTTTTGCTCCATAGTTCC | FG1 | 20.5 | CUFvAB28D19 | 3 |
|  |  | R: TTCATCAGGATCCAGAAGTCC |  |  |  |  |
| F3H | gene STS | F: GGTTGAAGGCACTGTCCATTA | FG1 | 40.6 | CUFvAB07G13 | 4 |
|  |  | R: GTCACAATCTCGCGCCAATCCT |  |  |  |  |
| EMFn182 | SSR | F: GCAACAAAGGAGGTTAGAGTCG | FG1 | 43.9 | CUFvAB31E17 | 3 |
|  |  | R: TGGTGAGTGCTCATTGTTCC |  |  | CUFvAB31E23 |  |
| EMFv185 | SSR | F: TCCAACAATGATTTCTCTCTGG | FG1 | 49.2 | CUFvAB35B13 | 5 |
|  |  | R: ATCGTGGTTTACTCCTGTGG |  |  |  |  |
| EMFv143 | SSR | F: ACAATTGGAGCGACACCTTC | FG1 | 49.2 | CUFvAB02A09 | 5 |
|  |  | R: CCGCGGTTTGATGAGTAAAA |  |  |  |  |
| EMFn128 | SSR | F: CATCAACATTCACATGAATTTACC | FG1 | 49.2 | CUFvAB42L24 | 3 |
|  |  | R: CGGCGGATCTAGTTTTGAGG |  |  | CUFvAB42O04 |  |
| CFVCT004 | SSR | F: ATGAGTGGTGCTGGGGTAGT | FG1 | 50.5 | CUFvAB26C14 | 6 |
|  |  | R: CCCATCTTCGCTTCACTCA |  |  | CUFvAB32F19 |  |
| EMFv164 | SSR | F: AAGTTGGCTGCCTATGGTGT | FG1 | 74.1 | CUFvAB14K20 | 5 |
|  |  | R: GGTGACTGACAGGGACCACT |  |  |  |  |
| EMFn148 | SSR | F: TTACCTGCACAGAAACAACG | FG2 | 7.6 | CUFvAB44G19 | 3 |
|  |  | R: CAACTTCCTCCTCACTCACC |  |  |  |  |
| CFVCT025 | SSR | F: CCTTCACTATGCTGCTCCAA | FG2 | 13.6 | CUFvAB17M15 | 5 |
|  |  | R: GAGGTTCCCTGTGCATTTGT |  |  |  |  |
| ARSFL012 | SSR | F: GCGGAACCAAGCCAATAAGATG | FG2 | 15.8 | CUFvAB08P13 | 7 |
|  |  | R: GCGACCACGACAGTTTCTCACTCT |  |  | CUFvAB31A05 |  |
| ADH | SNP | F: CCAAGGTACACATTCTTTTTTTC | FG2 | 17.9 | CUFvAB04F12 | 8 |
|  |  | R: CTCTCCACAATCCTGAATTTTAG |  |  |  |  |
| EMFv031 | SSR | F: AGGTTTTGTTTGCTTTTT | FG2 | 27.6 | UCFvAB21O16 | 5 |
|  |  | R: GTGGTTTGCTACTACTTGAT |  |  |  |  |
| EKO | gene STS | F: AGAAGAAACGAATTGCTTCAGG | FG2 | 28.9 | CUFvAB03P21 | 6 |
|  |  | R: TTGGTCCATTATCAGTGTC |  |  |  |  |
| EMFn134 | SSR | F: TGATTCTTTGAAAGGCTTTGG | FG2 | 50.1 | CUFvAB40B03 | 3 |
|  |  | R: AAAACAACCCCCTCTCATCC |  |  | CUFvAB40K09 |  |
| DFR | gene STS | F: CACCGGAGTGTTTCATGTCG | FG2 | 70.5 | CUFvAB06L09 | 4 |
|  |  | R: AACCTCCGAACTGTCTTTGC |  |  |  |  |
| EMFv003 | SSR | F: CTCTGATTCTTCTTCGTCCACCAT | FG2 | 74.1 | CUFvAB31I23 | 3 |
|  |  | R: TCCCCAGAGAATTAAACAGTCGTA |  |  | CUFvAB42L09 |  |
|  |  |  |  |  | CUFvAB44A05 |  |
| EMFv183 | SSR | F: TAGCCTTGCCAATCAAATCC | FG2 | 88.2 | CUFvAB07P21 | 3 |
|  |  | R: CCACTTTGGAAGACCAGTGC |  |  |  |  |
| EMFv029 | SSR | F: TACTATTGAAGAAACTCCTACTGA | FG3 | 2.4 | CUFvAB45I03 | 5 |
|  |  | R: TCTTTGATCTGCTTCCACCTT |  |  |  |  |
| UDF017 | SSR | F: GGACGTTCCACATCCGTAGA | FG3 | 24.8 | CUFvAB19B09 | 2 |
|  |  | R: CGGTGGAGATGTGATTTTATGA |  |  |  |  |
| EMFn034 | SSR | F: GCCTCAAAGATCACTCATTTCC | FG3 | 42.2 | CUFvAB22O08 | 1 |
|  |  | R: TCTTCATCTCTTTCAACCTCAAA |  |  |  |  |
| CFVCT035 | SSR | F: CCGGTCAAAACACCAAAACT | FG3 | 43.2 | CUFvAB40P19 | 6 |
|  |  | R: CTGGAAAGGAAACGATTGGA |  |  | CUFvAB38P20 |  |
| CFVCT022 | SSR | F: ATAATCCCCTTCCCATCACC | FG3 | 46.2 | CUFvAB1M19 | 6 |
|  |  | R: GACATTTTTAGCGGGTGGTC |  |  | CUFvAB5O19 |  |
|  |  |  |  |  | CUFvAB10M19 |  |
| APX | gene STS | F: GCATATCTGGTCTCGATTCTGC | FG3 | 49.5 | CUFvAB13L21 | 9 |
|  |  | R: CCCATAGAAACAACACCTACTGC |  |  |  |  |
| CFVCT007 | SSR | F: AAACAACACTCAAAGTACCGATCT | FG3 | 52.1 | CUFvAB05I20 | 6 |
|  |  | R: TTCAACATGCAAGCCTGATA |  |  | CUFvAB44P15 |  |
| CFVCT012 | SSR | F: GGAAAGGTTGGTACGACAGG | FG3 | 82.2 | CUFvAB34M13 | 6 |
|  |  | R: CCCCTCCCACAATATCTCTT |  |  | CUFvAB36J2 |  |
| EPpCU9642 | EST | F: TTCAGTTGGCAGATCCTGTG | FG3 | 0,0-26,7* | CUFvAB33F02 | 8 |
|  |  | R: TGCTGAGACCCTTCCAATTT |  |  | CUFvAB21C08 |  |
| EFaUF6868 | EST | F: GCTCTTCCAGGTCGAGTACG | FG3 | 53,9-73,8* | CUFvAB15C01 | 8 |
|  |  | R: GTTTCCACTTGGGCAGTTGT |  |  | CUFvAB15M01 |  |
|  |  |  |  |  | CUFvAB38A09 |  |
| UDF007 | SSR | F: TGAGTAAATGATGCAACCCAGA | FG4 | 24.7 | CUFvAB33B02 | 2 |
|  |  | R: GCTTGAGTATGTATTTGAGTGTATGTG |  |  |  |  |
| UFFxa01H05 | EST-SSR | F: GGGAGCTTGCTAGCTAGATTTG | FG4 | 25 | UCFvAB14K12 | 3 |
|  |  | R: AGATCCAAGTGTGGAAGATGCT |  |  |  |  |
| CFVCT014 | SSR | F: GGCACCACGGATTTCAAGTA | FG4 | 25.3 | CUFvAB15P07 | 6 |
|  |  | R: TGTTGCGTTTTCAAGCTCAC |  |  | CUFvAB32L14 |  |
| EMFv180 | SSR | F: AAGTTGGCTGCCTATGGTGT | FG4 | 26 | CUFvAB14K20 | 3 |
|  |  | R: GGTGACTGACAGGGACCACT |  |  | CUFvAB33E08 |  |
| EMFvi136 | SSR | F: GAGCCTGCTACGCTTTTCTATG | FG4 | 46.1 | CUFvAB14I04 | 10 |
|  |  | R: CCTCTGATTCGATGATTTGCT |  |  |  |  |
| CEL1 | gene STS | F: TACATCATGGTTGTGTCTGTCC | FG4 | 68.3 | CUFvAB23O15 | 9 |
|  |  | R: TCCAGAAAGCCTGATTCTAAGG |  |  |  |  |
| UDF020 | SSR | F: ATGTCCATTTGCCGACATTT | FG4 | 26 | CUFvAB32F24 | 2 |
|  |  | R: GGGTTTATTGGGTTGGGTTT |  |  |  |  |
| UDF008 | SSR | F: TGTTTGCGTGCCGATTATTA | FG4 | 77.5 | CUFvAB25P04 | 2 |
|  |  | R: TTAGCTCGCGTAAACTTCAGA |  |  |  |  |
| EFvVB2179 | EST | F: ATCTGCGTGACAATGCAAAG | FG4 | 0,0-26,0* | CUFvAB06A13 | 8 |
|  |  | R: AAGAGCCTTCAGTTGCTCCA |  |  | CUFvAB40C09 |  |
| EFvVB1231 | EST | F: CCAACTGTGACATCCACGAC | FG4 | 26,0-46,1* | CUFvAB41P20 | 8 |
|  |  | R: GCTGTCACGCAGAAAATCAA |  |  | CUFvAB08A14 |  |
| EMFvi108 | SSR | F: GGACCCCAAAACATTGAATAAA | FG5 | 7.7 | CUFvAB03H03 | 10 |
|  |  | R: GAAGAGGGGAGGAGCAATAAAT |  |  | CUFvAB30G01 |  |
|  |  |  |  |  | CUFvAB41E14 |  |
| ANS | gene STS | F: AAGGAGAAGTATGCCAATGACC | FG5 | 9.7 | CUFvAB13A02 | 4 |
|  |  | R: CTCCCTTCTTCTAATCCCAAGC |  |  |  |  |
| RAN | gene STS | F: TTCACCAAAACAACCAG | FG5 | 15.3 | CUFvAB21J01 | 4 |
|  |  | R: GTACCCATCACCCCACTC |  |  |  |  |
|  |  |  |  |  |  |  |
| CEL2 | gene STS | F: GGTGTTCAGACCCTTGTTGC | FG5 | 29.2 | CUFvAB06C16 | 9 |
|  |  | R: ATGCTTACCAGCTTTACCTTGC |  |  |  |  |
| EMFn110 | SSR | F: GACGCTTCGGAGACTGAGG | FG5 | 36.4 | CUFvAB44G07 | 3 |
|  |  | R: CCCCCTTAAAAATAATTAAATCTCC |  |  |  |  |
| CFVCT024 | SSR | F: CCCCGGAAAGCTGGTAAATA | FG5 | 47.7 | CUFvAB07E15 | 6 |
|  |  | R: TCTTCAAATTGTGGCTGCAT |  |  | CUFvAB14P15 |  |
|  |  |  |  |  | CUFvAB43B11 |  |
| CFVCT016 | SSR | F: CACAACGGAGTACACGGCTA | FG5 | 68.5 | CUFvAB20O01 | 6 |
|  |  | R: TCAGATTCACCTCGGACCTC |  |  | CUFvAB20O11 |  |
| CFVCT003 | SSR | F: TCGTCGATCTCTAAAACAAGGTC | FG5 | 69.2 | CUFvAB42K22 | 6 |
|  |  | R: ACCACCACCCATTCTGAGTT |  |  |  |  |
| EMFvi018 | SSR | F: CAAACATGGAAGGAAAAGAAGG | FG5 | 72.5 | CUFvAB32N02 | 10 |
|  |  | R: GTCAGAGAGACCCATCTGAACC |  |  |  |  |
| EFvVB2013 | EST | F: GTGCAGTTGCCAAAGGAGC | FG5 | 50,4-72,5* | CUFvAB08J20 | 8 |
|  |  | R: AGCTGGGTTTGCTGCTT |  |  |  |  |
| ARSFL-007 | SSR | F: GCGCGCATAAGGCAACAAAG | FG6 | 0 | CUFvAB19N24 | 7 |
|  |  | R: GCGAATGGCAATGACATCTTCTCT |  |  | CUFvAB30F04 |  |
| EPpCU1830 | EST | F: TGATGCAATTGGCACAAAGC | FG6 | 14.1 | CUFvAB14H08 | 8 |
|  |  | R: CCTATCACCACTTACTTCACTGC |  |  |  |  |
| PGLM | gene STS | F: AAGAGGACCTTGCAAAGTTACG | FG6 | 24.5 | CUFvAB14N12 | 9 |
|  |  | R: GTGTCTTGATTGCTTCAACAGC |  |  |  |  |
| EMFn117 | SSR | F: ATCGGATCAACAAGCAAAGC | FG6 | 30.9 | CUFvAB31015 | 3 |
|  |  | R: ATGGATGAGGGGAGAAGAGG |  |  | CUFvAB33M21 |  |
| CFVCT002 | SSR | F: GATGCTAAGTAGCCGCACCT | FG6 | 57.9 | CUFvAB44H23 | 6 |
|  |  | R: TGGACCTTTTTGGGTATGGT |  |  |  |  |
| CFVCT010 | SSR | F: TGACAGAGACAAATGCATCACA | FG6 | 57.9 | CUFvAB42K10 | 6 |
|  |  | R: CGACGTTTGCCCTCTTTCTA |  |  |  |  |
| CFVCT036 | SSR | F: AAGCTTTTCCCACCGAGAAT | FG6 | 59.2 | CUFvAB22L24 | 6 |
|  |  | R: CCAACAATCCGATGCCTAAG |  |  | CUFvAB34C03 |  |
| CFVCT017 | SSR | F: GCATCTCCAAAGCTCTCACG | FG6 | 71.5 | CUFvAB16L06 | 6 |
|  |  | R: GCCTAAACCAAACCCAAAATC |  |  | CUFvAB16K18 |  |
| UFFxa01E03 | EST-SSR | F: ACCCCATCTTCTTCAAATCTCA | FG6 | 114.7 | CUFvAB33P23 | 3 |
|  |  | R: GACAAGGCCAGAGCTAGAGAAG |  |  | CUFvAB33H10 |  |
| EFvVB2119 | EST | F: GCTCGAGCTGATTACGATTACC | FG6 | 22,5-43,2* | CUFvAB29F20 | 8 |
|  |  | R: TAAAGGACCCATCAGAGAAACG |  |  |  |  |
| EMFv021 | SSR | F: TCATTTTTCAGGGCCACGGGTAGA | FG7 | 16.2 | CUFvAB06A15 | 5 |
|  |  | R: GTGGTGGTTGAGGCAGTGGAGGAT |  |  | CUFvAB24E18 |  |
| CFVCT026 | SSR | F: CGCGTCAAAGATACACGAGA | FG7 | 17.5 | CUFvAB45M14 | 6 |
|  |  | R: GGCTTCCGATCGATATTCAA |  |  |  |  |
| CHS | gene STS | F: TATCCCGACTACTACTTC | FG7 | 17.5 | CUFvAB01G08 | 4 |
|  |  | R: GCTGGCCCCATTCCTTAA |  |  |  |  |
| EPpCU2875 | EST | F: AACTCAGAGACATATCTGCACAGG | FG7 | 20.3 | CUFvAB24G05 | 8 |
|  |  | R: AAGTTGAAGCGGTCTTCATAGG |  |  |  |  |
| EMFn201 | SSR | F: CAGCTCAGAAAAGCTCACAGC | FG7 | 20.6 | CUFvAB07B11 | 3 |
|  |  | R: TAGAACGCCAATCACAAACC |  |  |  |  |
| CHI | gene STS | F: AAGACGGCCGAGGAGTTG | FG7 | 28.2 | CUFvAB40D22 | 4 |
|  |  | R: CGTCAGCGGTAGTATCATTGTC |  |  |  |  |
| CFVCT019 | SSR | F: ATGGCGTCATACCATCATCA | FG7 | 30.9 | CUFvAB05H08 | 6 |
|  |  | R: CTGCGGTTTGAGAGGACTTC |  |  | CUFvAB13K05 |  |
|  |  |  |  |  | CUFvAB25K16 |  |
| EPpCU9223 | SSR | F: AACAGAGCCAAGCTTATGCAG | FG7 | 44.5 | CUFvAB13D14 | 8 |
|  |  | R: TTTCTGCGCAACCGCATC |  |  |  |  |
|  |  |  |  |  |  |  |
| EMFv023 | SSR | F: AATTACCGAGCCTCCCACACTA | FG7 | 64.1 | CUFvAB24I04 | 5 |
|  |  | R: CAGCGCTAAAGCGGTTGC |  |  |  |  |
| EFaUF7084 | EST | F: CAGAAGAGGTTCAAGTTCC | FG7 | 63,4-81,00* | CUFvAB18K01 | 8 |
|  |  | R: ACACCATAGCAAGCCCTG |  |  |  |  |

*Markers bin mapped in the FV×FN population and mapping interval of the bin to which they were located is given.

†References for primer sequences used to amplify loci.

1. Sargent DJ, Davis TM, Tobutt KR, Wilkinson MJ, Battey NH, Simpson DW: **A genetic linkage map of microsatellite, gene specific and morphological markers in diploid *Fragaria***. Theor Appl Genet 2004, 109: 1385–1391.

2. Cipriani G, Testolin R: **Isolation a characterisation of microsatellite loci in *Fragaria***. Mol Ecol Notes 2004, 4:366–368.

3. Sargent DJ, Clarke J, Simpson DW, Tobutt KR, Arús P, Monfort A, Vilanova S, Denoyes-Rothan B, Rousseau M, Folta KM, Bassil NV, Battey NH: **An enhanced microsatellite map of diploid *Fragaria***. *Theoretical and Applied Genetics* 2006, 112:1349-1359.

4. Deng C, Davis TM: **Molecular identification of the yellow fruit color (c) locus in diploid strawberry: a candidate gene approach**. *Theoretical and Applied Genetics* 2001, 103:316–322.

5. Hadonou AM, Sargent DJ, Wilson F, James CM, Simpson DW: **Development of microsatellite markers in *Fragaria*, their use in genetic diversity analysis and their potential for genetic linkage mapping**. *Genome* 2004, 47:429–438.

6. Monfort A, Vilanova S, Davis TM, Arús P: **A new set of polymorphic simple sequence repeat (SSR) markers from a wild strawberry (*Fragaria vesca*) are transferable to other diploid Fragaria species and to *Fragaria ×ananassa***. *Molecular Ecology Notes* 2006, 6: 197-200.

7. Ashley MV, Wilk JA, Styan SMN, Craft KJ, Jones KL, Feldheim KA, Lewers KS, Ashman TL: **High variability and disomic segregation of microsatellites in octoploid *Fragaria virginiana* Mill. (Rosaceae)**. *Theoretical and Applied Genetics* 2003, 107:1201–1207.

8. Vilanova S, Sargent DJ, Arús P, Monfort A: **Synteny conservation between two distantly-related Rosaceae genomes: *Prunus* (the stone fruits) and *Fragaria* (the strawberry)**. *BMC Plant Biology* 2008, 8:67.

9. Sargent DJ, Rys A, Nier S, Simpson DW, Tobutt KR: **The development and mapping of functional markers in *Fragaria* and their transferability and potential for mapping in other genera**. *Theoretical and Applied Genetics* 2007, 114: 373-384.

10. Sargent DJ, Hadonou AM, Simpson DW: **Development and characterisation of polymorphic microsatellite markers from *Fragaria viridis*, a wild diploid strawberry**. *Molecular Ecology Notes* 2003, 3: 550–552.
